# Enhancing chemosensitivity to gemcitabine via RNA interference targeting the catalytic subunits of protein kinase CK2 in human pancreatic cancer cells

**DOI:** 10.1186/1471-2407-10-440

**Published:** 2010-08-19

**Authors:** Jan N Kreutzer, Maria Ruzzene, Barbara Guerra

**Affiliations:** 1 Department of Biochemistry and Molecular Biology, University of Southern Denmark, Odense, Denmark; 2 Department of Biological Chemistry and Venetian Institute of Molecular Medicine, University of Padova, Padova, Italy

## Abstract

**Background:**

Pancreatic cancer is a complex genetic disorder that is characterized by rapid progression, invasiveness, resistance to treatment and high molecular heterogeneity. Various agents have been used in clinical trials showing only modest improvements with respect to gemcitabine-based chemotherapy, which continues to be the standard first-line treatment for this disease. However, owing to the overwhelming molecular alterations that have been reported in pancreatic cancer, there is increasing focus on targeting molecular pathways and networks, rather than individual genes or gene-products with a combination of novel chemotherapeutic agents.

**Methods:**

Cells were transfected with small interfering RNAs (siRNAs) targeting the individual CK2 subunits. The CK2 protein expression levels were determined and the effect of its down-regulation on chemosensitization of pancreatic cancer cells was investigated.

**Results:**

The present study examined the impact on cell death following depletion of the individual protein kinase CK2 catalytic subunits alone or in combination with gemcitabine and the molecular mechanisms by which this effect is achieved. Depletion of the CK2α or -α' subunits in combination with gemcitabine resulted in marked apoptotic and necrotic cell death in PANC-1 cells. We show that the mechanism of cell death is associated with deregulation of distinct survival signaling pathways. Cellular depletion of CK2α leads to phosphorylation and activation of MKK4/JNK while down-regulation of CK2α' exerts major effects on the PI3K/AKT pathway.

**Conclusions:**

Results reported here show that the two catalytic subunits of CK2 contribute differently to enhance gemcitabine-induced cell death, the reduced level of CK2α' being the most effective and that simultaneous reduction in the expression of CK2 and other survival factors might be an effective therapeutic strategy for enhancing the sensitivity of human pancreatic cancer towards chemotherapeutic agents.

## Background

Pancreatic cancer is one of the most aggressive human solid tumors which rapidly grows and metastasizes, representing one of the leading causes of cancer-related death in developed countries [1,2]. Current treatment regimens for patients with pancreatic cancer that are not suitable for surgical resection are still not effective, due to low response rates and a 5-6 months median survival [1,2]. Over the past decades, multiple randomized trials have sought to improve the outcome of patients with advanced pancreatic cancer including treatment with platinum agents, taxanes and topoisomerase inhibitors [3]. Moreover, there has been considerable interest in combining gemcitabine (2',2'-difluoro 2'-deoxycytidine), the first-line treatment option, with ionizing radiation and a variety of other agents that exert various mechanisms of action. Based on the acquired knowledge on the molecular biology of this disease [4], new approaches (i.e. combination therapy where chemotherapeutic agents are administered with compounds, such as inhibitors, targeting pro-survival proteins and protein kinases) in pancreatic cancer treatment have recently emerged [5].

Protein kinase CK2 is a serine/threonine kinase, highly conserved and ubiquitously expressed in eukaryotic cells. Traditionally, CK2 has been described as a constitutively active enzyme composed of two catalytic α and/or α' and two regulatory β subunits [6-8] but mounting evidence has recently modified the classical view of CK2 as a stable tetrameric complex, revealing that the individual CK2 subunits may be asymmetrically distributed and exert independent functions in cells [9]. The high degree of conservation of CK2 suggests that this enzyme might be essential for cell viability. Indeed, complete suppression of the CK2 α- or β-subunits leads to embryonic lethality in mice while knockout of CK2α' results in viable offspring but leads to sterility in male mice due to defective spermatogenesis [10-12]. Considerable information on the role of CK2 in various diseases has been gained in recent years [8] making it a promising therapeutic target particularly for the treatment of cancer [13]. CK2 has been involved in neurodegenerative disorders where a number of structural proteins and enzymes involved in various functions of the nervous system have been identified as CK2 substrates, in inflammatory processes, in diseases of the vascular system, in various parasites- and viral-related diseases [8]. Overexpression of CK2 has been documented in a number of cancers where deregulation of intracellular signaling pathways and association with the aggressiveness of the tumor have been observed [13]. Cooperative increase in tumorigenesis in cells co-expressing oncogenes and CK2 has also been reported demonstrating a critical role of CK2 in the progression of malignancies [6,13].

Recently, the development of a systematic approach by which over 600 kinases were individually silenced by small interfering RNAs (siRNAs) revealed that down-regulation of the CK2 α-subunit increases the sensitivity of pancreatic cancer cells to gemcitabine [14]. Similarly, the pharmacological inhibition of CK2 has been shown to counteract the apoptosis resistance of a T lymphoblastoid cell line [15].

In this study, we aimed to closely investigate the role of protein kinase CK2 in human pancreatic cancer cells highly resistant to chemotherapeutic treatment. We report evidence that the cellular depletion of CK2α and -α' by siRNAs markedly enhances the sensitivity of cancer cells to gemcitabine treatment. Moreover, we show that the individual CK2 catalytic subunits contribute differently to the modulation of intracellular survival pathways resulting in distinct cellular responses towards drug treatment.

## Methods

### Cell culture and treatments

The pancreatic ductal adenocarcinoma cell lines Mia PaCa-2, PANC-1, BxPC-3 and Capan-1 were purchased from the American Type Culture Collection and maintained under the conditions recommended by the supplier. Photographs of the cells were taken under a phase contrast microscope (Leica, DM IRB, Germany). Silencing of CK2 expression was achieved by transfection of cells with siRNA duplexes directed against the individual catalytic subunits (ON-TARGET plus SMARTpools, Dharmacon, CO, USA). Cells were transfected with Lipofectamine 2000 (Invitrogen, CA, USA) for up to 96 h following the manufacturer's recommendations. Gemcitabine (Eli Lilly, Germany) treatment was performed 24 h after transfection for 72 h. Where indicated, cells were incubated for 72 h with the broad range caspase inhibitor, z-Val-Ala-Asp-fluoromethyl-ketone [zVAD(OMe)-fmk, Calbiochem, CA, USA] at a concentration of 5 μM and the cathepsin B inhibitor, z-Phe-Ala-fmk (zFA-fmk, Calbiochem) at a concentration of 85 μM.

### Determination of cell viability and proliferation

The WST-1 viability assay (Roche, Germany) was performed in 96-well plates. Twenty-four hours after seeding, cells were treated with various concentrations of gemcitabine for 72 h. WST-1 reagent was added to the cells according to the manufacturer's instructions. Conversion of the WST-1 reagent into formazan salts by metabolically active cells was measured 2 h after addition of the reagent in a microtiter plate reader (Perkin-Elmer, MA, USA). The Cell Proliferation Assay (Calbiochem) was performed in 96-well tissue culture plates. After 72 h treatment, cells were labeled with 5-bromo-2'-deoxyuridine (BrdU) for 3 h. Cells were then fixed, DNA was denatured and cells were subsequently incubated with a peroxidase-conjugated anti-BrdU antibody. The immune complexes were revealed in a microtiter plate reader by the subsequent substrate reaction according to the manufacturer's instructions.

### Flow cytometry

Cells were collected after various treatments by trypsinization, washed with PBS and fixed overnight in 70% ethanol at -20°C. For cell cycle analysis and determination of cell death (i.e. sub-G1 region), cells were incubated for 30 min in the dark with 20 μg/ml propidium iodide (Sigma, MO, USA) and 40 μg/ml RNase A (Roche) in PBS. Cells were analyzed on a FACS-Calibur flow cytometer (Becton Dickinson, CA, USA). The acquired data were analyzed by Cell Quest Pro Analysis software (Becton Dickinson). For each measurement, 10,000 cells were analyzed. The method allows the quantification of cells with reduced DNA (i.e. in late apoptosis or necrosis).

### Western blot analysis and protein kinase assays

Cell lysates were prepared as described in [16]. Proteins were detected by probing Western blot membranes with the following antibodies: mouse monoclonal anti-CK2α/α', mouse monoclonal anti-CK2β (both from Calbiochem); mouse monoclonal anti-β-actin (Sigma); rabbit polyclonal anti-CK2α' obtained by immunizing rabbits with a specific peptide sequence of human CK2α': ^334^SQPCADNAVLSSGTAAR^350^; mouse monoclonal anti-poly(ADPribose)polymerase (PARP), mouse monoclonal anti-mTOR, mouse monoclonal anti-PDK1, mouse monoclonal anti-AKT, mouse monoclonal anti-GSK3β (all from BD Biosciences, CA, USA); rabbit polyclonal anti-p44/42MAPK, rabbit monoclonal anti-phospho-p44/42MAPK (T202/Y204), mouse monoclonal anti-phospho-p38MAPK (T180/Y182), rabbit polyclonal anti-MKK4, rabbit monoclonal anti-c-Jun, rabbit polyclonal anti-phospho-c-Jun (S63), mouse monoclonal anti-phospho-p70 S6 kinase (T389), rabbit polyclonal anti-phospho-AKT (T308), mouse monoclonal anti-phospho-AKT (S473), rabbit polyclonal anti-phospho-GSK3β (S9) (all from Cell Signaling Technology, MA, USA); rabbit polyclonal anti-p38MAPK, rabbit monoclonal anti-JNK, rabbit polyclonal anti-p70 S6 kinase, (all from Santa Cruz Biotechnology, CA, USA); rabbit polyclonal anti-phospho-JNK (T183, Y185, BioSource, CA, USA). Expression of major proteins of the autophagy machinery was analyzed by employing the autophagy antibody sample kit (Cell Signaling Technology). Rabbit polyclonal anti-phospho-AKT (S219) antibody was obtained as described in [17]. Protein-antibody complexes were visualized by a chemiluminescence Western blot detection system according to the manufacturer's instructions (CDP-Star, Applied Biosytems, CA, USA). Immunoprecipitation experiments were performed essentially as described in [18]. The activity of protein kinase CK2 was determined as reported in [16]. The activity of JNK was tested in a non-radioactive assay (SAPK/JNK assay kit, Cell Signaling Technology) following the manufacturer's recommendations in the absence or presence of 20 μM SP600125 inhibitor (Calbiochem).

### Statistical analysis

The two-tailed *t*-test (Student's *t*-test) was performed to evaluate the statistical significance of differences between the mean of two sets of data.

## Results

### Cellular response to gemcitabine of various pancreatic cancer cell lines

**Figure 1:**
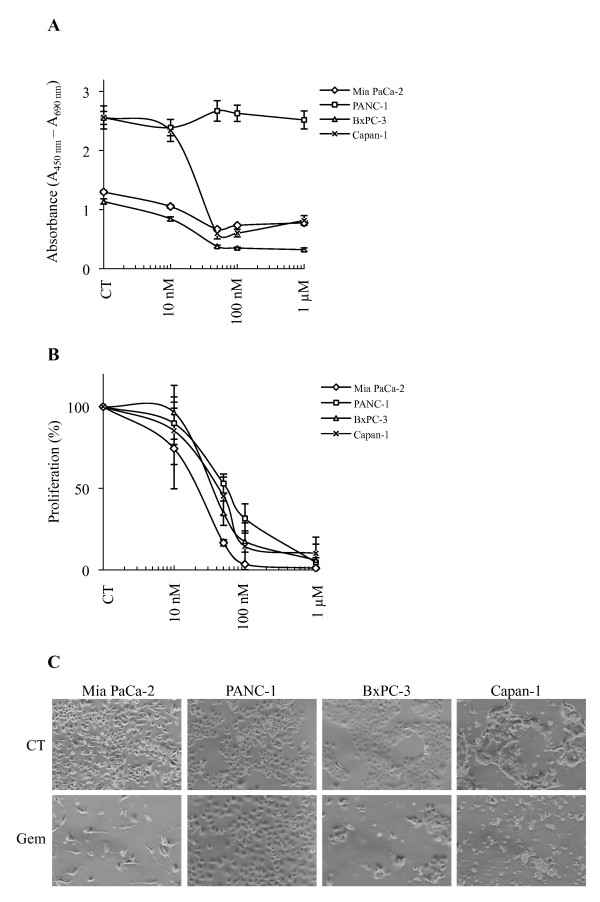
**Effect of gemcitabine on viability and proliferation of four human pancreatic cancer cell lines**. **A**. Cells were treated with the indicated concentrations of gemcitabine for 72 h. Proportions of viable cells measured by the WST-1 assay are shown in arbitrary units as a difference in absorbance measured at 450 nm and 690 nm (reference) wavelengths, respectively. **B**. Cell proliferation was determined by BrdU incorporation into genomic DNA. The results are expressed in percentage relative to the corresponding untreated controls. In both assays, three separate experiments were performed and data from one representative experiment [mean +/- standard deviation (STD) of four replicates] are shown. **C**. Phase contrast microscopy photographs of cells left untreated or incubated with 50 nM gemcitabine for 72 h. Pictures were originally captured at 400× magnification.

**Figure 2:**
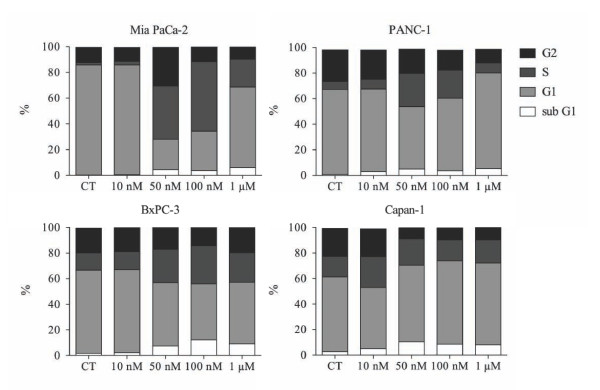
**Effect of gemcitabine on the cell cycle profile of human pancreatic cancer cell lines**. Cells were left untreated (CT) or incubated with increasing concentrations of gemcitabine (Gem) for 72 h. Fixed cells were analyzed by flow cytometry after propidium iodide staining. The amount of cells in the various phases of the cell cycle is indicated in percentage. Four separate experiments were performed obtaining similar results and data from one representative experiment are shown.

Dose-response experiments were performed measuring metabolically active cells in the absence and presence of gemcitabine at different concentrations for 72 h, respectively, (Figure 1A). 50 nM gemcitabine induced cytotoxicity in Mia PaCa-2, BxPC-3 and Capan-1 cells by reducing the viability to more than 50% while PANC-1 cells remained unaffected. The anti-proliferative effect of gemcitabine was also investigated by measuring BrdU incorporation into the newly synthesized DNA of replicating cells (Figure 1B). 50 nM gemcitabine exerted a major effect on Mia PaCa-2, BxPC-3 and Capan-1 cell lines as their proliferation decreased more than 50%, with respect to control experiments while the proliferation of PANC-1 cells decreased about 50%. This is also documented by the analysis of cells by phase contrast microscopy (Figure 1C). The response to gemcitabine treatment was also analyzed by flow cytometry (Figure 2). Cells were treated with various dosages of gemcitabine for 72 h prior harvesting. Incubation with 50 nM gemcitabine was sufficient to induce either G1/S- (Capan-1) or S phase cell cycle arrest (Mia PaCa-2, PANC-1 and BxPC-3) with respect to control cells (CT). Gemcitabine up to a concentration of 1 μM did not result in a significant percentage of cells with reduced DNA levels in any of the analyzed cell lines supporting the notion that pancreatic cancer cells are highly resistant to chemotherapeutic treatments as previously reported [19,20].

### Down-regulation of protein kinase CK2 enhances gemcitabine-induced cell death

**Figure 3:**
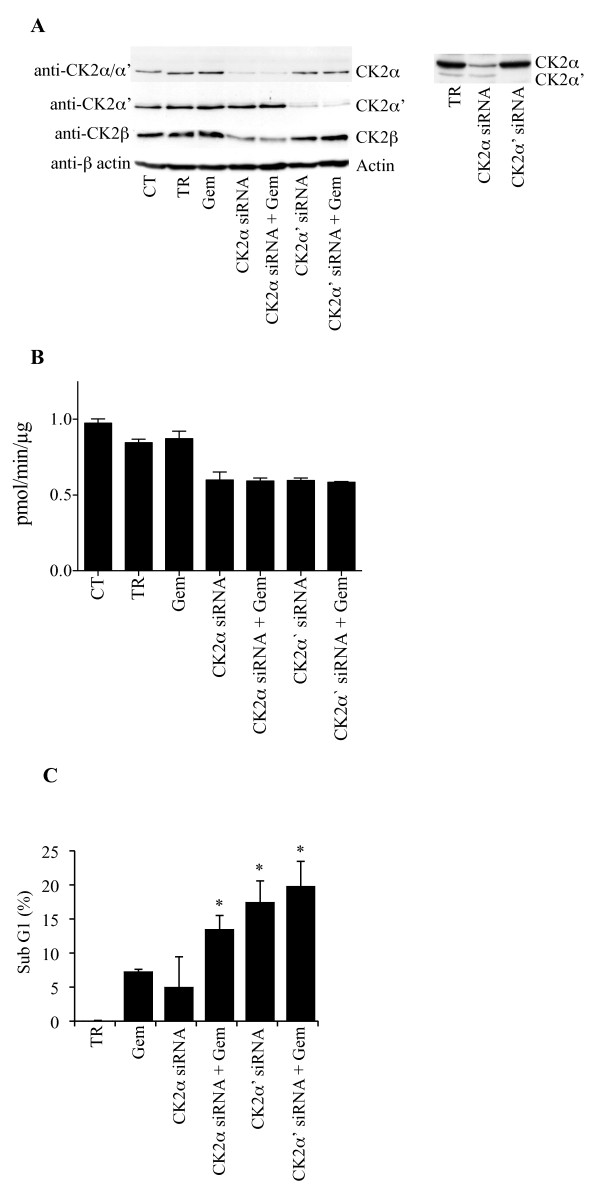
**Cellular depletion of the individual CK2 catalytic subunits enhances gemcitabine-mediated cell death**. **A**. PANC-1 cells were left untreated (CT), treated with transfection reagent (TR) or siRNAs against the catalytic α- and α'-subunits of CK2 for 96 h. One day after transfection, cells were cultured for 72 h in the presence of 50 nM gemcitabine. Total cell lysates (50 μg) were analyzed by Western blot using the indicated mouse monoclonal antibodies. β-actin was used as control for equal loading. Insert shows Western blot analysis (long autoradiographic exposure) of whole extracts from cells treated with transfection reagent, CK2α-siRNA or CK2α'-siRNA, using anti-CK2α/α' antibody. Data shown are representative of three independent experiments. **B**. Cells were treated as described in A. Total lysates (15 μg) were subjected to a radioactive CK2 kinase assay with a specific CK2 peptide substrate as described in [14]. The average +/- STD of three independent experiments are shown. **C**. Cells treated as described above were fixed, stained with propidium iodide and analyzed by flow cytometry. The fraction of dead cells expressed in percentage (average from four independent experiments +/- STD) is reported on the ordinate axis after subtraction of the average sub-G1 value (i.e. 17.5%) of cells treated with transfection reagent. Stars denote statistically significant differences in the percentage of cell death after treatment with CK2α-siRNA and gemcitabine or CK2α'-siRNA alone or in combination with gemcitabine as compared to the treatment with the sole gemcitabine (Student's *t*-test, P < 0.05). CK2α'-siRNA treatment was tested against CK2α'-siRNA in combination with gemcitabine (no statistically significant difference). Statistically significant difference was found when comparing gemcitabine treatment versus control (results not shown).

**Figure 4:**
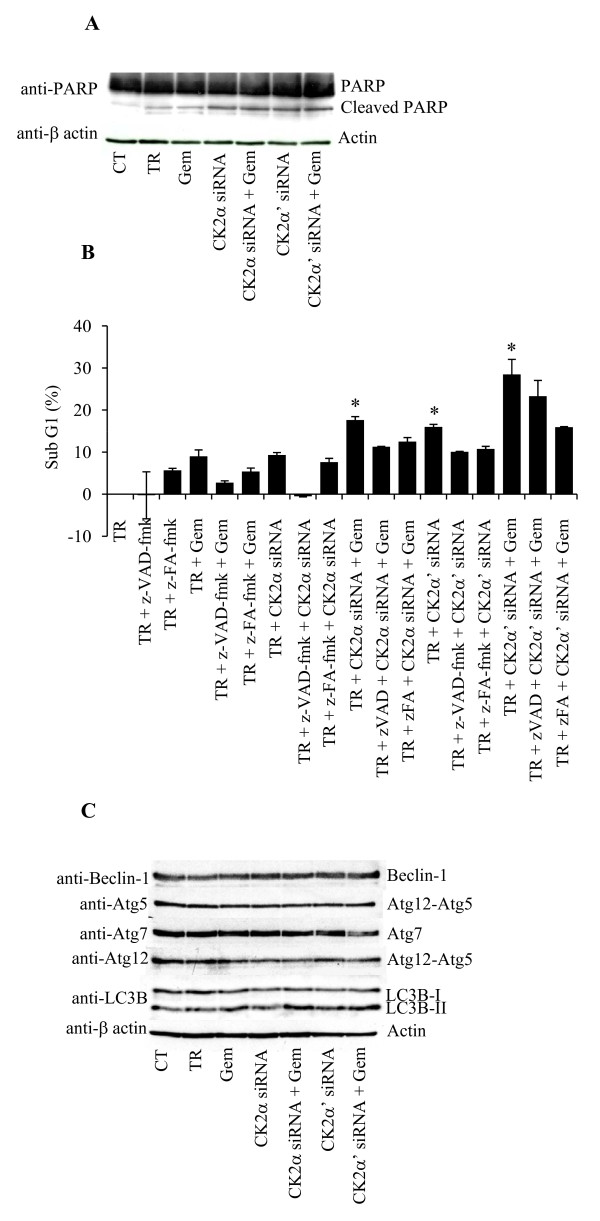
**Reduced CK2 levels in cells treated with gemcitabine result in apoptotic and necrotic cell death**. **A**. Whole lysates from cells treated as indicated in the figure were subjected to Western blot analysis of PARP cleavage. Full length PARP (116 kDa) and the corresponding cleavage product (85 kDa) are indicated. β-actin was used as control for equal loading. **B**. Cells treated as indicated below the bar graph were subjected to flow cytometry analysis following propidium iodide staining. The amount of cells in sub-G1 is reported in percentage after subtraction of the percentage of cell death in cells treated with transfection reagent. Average from three independent experiments +/- STD is shown. Stars denote statistically significant difference in the percentage of cell death after treatments with CK2α-siRNA and gemcitabine, CK2α'-siRNA or CK2α'-siRNA and gemcitabine as compared to the treatment with gemcitabine alone (Student's *t*-test, P < 0.03). Statistically significant difference was also found when comparing gemcitabine treatment versus control (results not shown). **C**. Whole lysates from cells treated as indicated were subjected to Western blot analysis with antibodies directed against a panel of proteins involved in autophagy. For Western blot analysis, three independent experiments were performed obtaining similar results and one representative experiment is shown.

To investigate the role of CK2 on the gemcitabine-mediated cellular response, we attempted to down-regulate its expression by employing siRNAs targeting the two individual α and α' subunits. The most gemcitabine-resistant cell line, PANC-1, was chosen in order to investigate whether CK2 suppression would significantly enhance the cytotoxic effect of gemcitabine. Protein expression analysis indicated that down-regulation of the individual CK2 subunits was visible after 4 days from the initial siRNA transfection (Figure 3A). Additionally, down-regulation of CK2α also caused decreased expression of CK2β, an effect that has been previously attributed to the tendency of CK2β to undergo rapid degradation in case of missing assembly with CK2α [21,22]. Cellular depletion of the individual CK2 catalytic subunits led to a 40% reduction of the CK2 kinase activity, whereas gemcitabine treatment did not affect the CK2 activity measured in the control experiment (Figure 3B). Next, flow cytometry analysis was conducted to measure cell death in response to gemcitabine treatment in CK2α or -α' subunit knockdown cells (Figure 3C). Treatment with 50 nM gemcitabine for 72 h led to 7% cell death similar to results shown in Figure 2. Gemcitabine treatment in CK2α or α'- reduced cells resulted in 14% and 20% cell death, respectively. Additionally, the sole depletion of CK2α' but not -α induced 17.5% cell death. Preliminary experiments conducted with scramble siRNA neither modified the percentage of control cells in sub-G1 nor the expression of the individual CK2 subunits (data not shown). Overall, these results show that the individual CK2 subunits do not affect cell death to the same extent and that the down-regulation of CK2α' and not -α is sufficient to kill cells. Moreover, they indicate that PANC-1 cells are sensitized to gemcitabine treatment following cellular depletion of the CK2α subunit by RNA interference. Next, it was investigated whether knockdown of CK2 in cells treated with gemcitabine would lead to an apoptotic type of cell death by looking at the cleavage of the caspase-3/caspase-7 substrate PARP. As shown in Figure 4A, the sole gemcitabine treatment resulted in a slight PARP cleavage which was comparable to the one corresponding to cells treated with the transfection reagent. Cellular depletion of the individual CK2 α- and α' subunits alone or in combination with gemcitabine resulted in a slightly higher induction of PARP cleavage. To further clarify the type of cell death induced by the treatments described above, flow cytometry analysis of propidium iodide-stained cells was performed in the presence or absence of a broad-range caspase inhibitor zVAD-fmk or cystein cathepsin inhibitor zFA-fmk as indicated in Figure 4B. The toxicity of gemcitabine in cells depleted of CK2α and -α', respectively, was reduced when cells were treated with zVAD-fmk or zFA-fmk indicating that the inhibitors partially prevented the toxic effects induced by the indicated treatments (Figure 4B). Western blot analysis of cell lysates performed by employing a panel of antibodies directed against proteins involved in autophagy excluded the involvement of this catabolic process in the observed cell death (Figure 4C). Overall, these results suggest that both the mitochondrial and lysosomal death pathways are activated and that the combination of gemcitabine and siRNAs against the individual CK2 subunits induces both apoptotic and necrotic types of cell death.

### Down-regulation of the CK2 catalytic subunits negatively affects survival pathways in gemcitabine-treated cells

**Figure 5:**
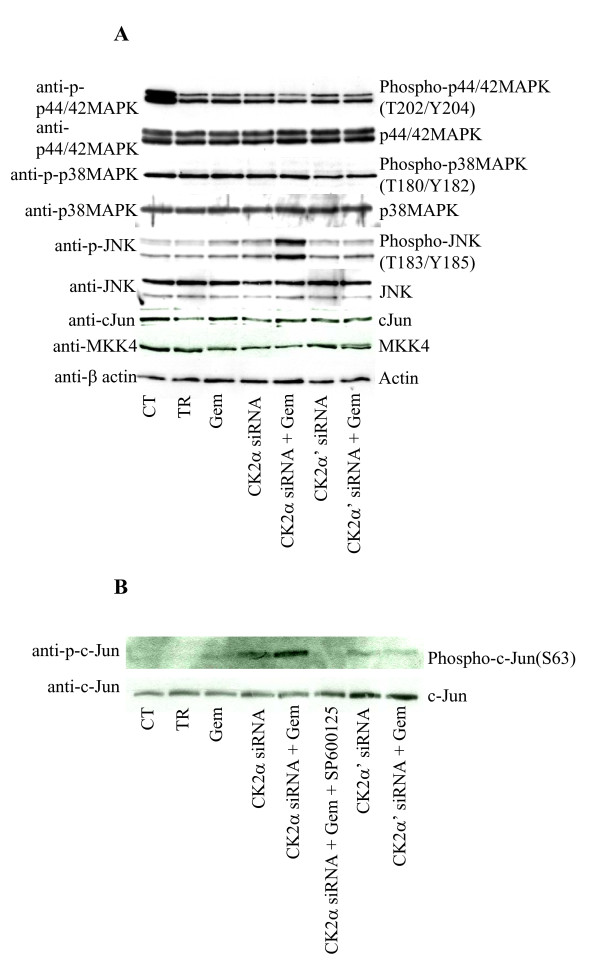
**Effect of gemcitabine on the MAPK pathway in CK2 subunits-depleted human PANC-1 cells**. **A**. Total cellular proteins (80 μg) from exponentially growing cells treated as indicated in the figure were subjected to Western blot analysis with antibodies directed against the proteins or their phosphorylated form as indicated. β-actin was applied as control for equal loading. p44/42MAPK: the upper band is p44MAPK, the lower one is p42MAPK. **B**. Whole lysates (1 mg) from cells treated as indicated in the figure were subjected to a non-radioactive kinase activity assay in the presence of a GST-c-Jun fusion protein linked to glutathione agarose beads. The precipitated complex was subjected to a phosphorylation assay as described in the materials and methods section. Where indicated, the JNK inhibitor SP600125 was used at a concentration of 20 μM. The protein and phosphorylation levels of c-Jun were detected by Western blot with the indicated antibodies. Representative results from three independent experiments are shown.

To determine the potential involvement of survival proteins in the mechanism of cell death, the expression and/or phosphorylation levels of the major protein kinases belonging to the family of mitogen-activated protein kinases namely p44/42MAPK, p38MAPK and Jun-amino-terminal kinase (JNK) were analyzed (Figure 5A). Cellular depletion of the individual CK2 catalytic subunits either alone or in combination with gemcitabine did not affect the expression levels and/or the phosphorylation status of p44/42MAPK and p38MAPK. Interestingly, down-regulation of CK2α but not -α' in combination with gemcitabine, caused a marked increase in JNK phosphorylation when compared to cells left untreated, treated with transfection reagent or with CK2α-siRNA. Gemcitabine treatment caused a slight increase in the phosphorylation of JNK which was comparable to the one relative to cells transfected with CK2α-siRNA, CK2α'-siRNA or CK2α'-siRNA and gemcitabine. To further demonstrate that in cells depleted of CK2α, gemcitabine-induced cell death was mediated by the JNK signaling pathway, a non-radioactive kinase assay was performed where the activity of endogenous JNK was tested against a c-Jun (a downstream target of JNK [23,24]) fusion protein linked to agarose beads. By employing a phospho-specific antibody directed against c-Jun, the activation of JNK following the above indicated treatment was verified (Figure 5B). Phosphorylation of c-Jun was blocked by the addition of the JNK inhibitor SP600125 (control assay, [25]). Except in cells depleted of CK2α and treated with gemcitabine, a similar activation of JNK was not observed with any other indicated treatment. Overall, these results support the notion that gemcitabine-induced cell death in cells depleted of CK2α is specifically mediated through the JNK signaling pathway in the PANC-1 cell line.

**Figure 6:**
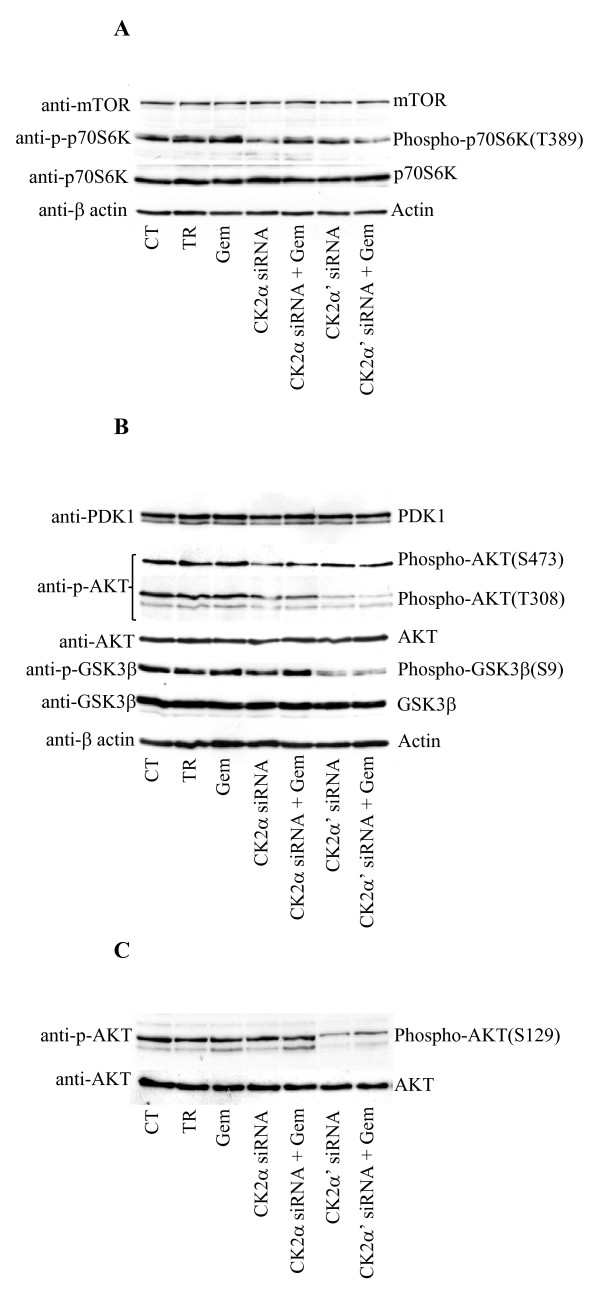
**Reduced levels of CK2 affect the pro-survival PI3K/AKT and mTOR signaling pathways in PANC-1 cells**. **A**. Western blot analysis of mTOR signaling pathway-related proteins in PANC-1 cells treated as indicated in the figure. **B**. Total lysates (80 μg) from cells treated as shown in the figure, were subjected to Western blot analysis with the indicated antibodies against PI3K/AKT signaling pathway-related proteins. β-actin was used as control for equal loading. **C**. Whole lysates from cells treated as indicated in the figure were subjected to Western blot analysis with the indicated antibodies. Data shown are representative of three independent experiments.

Next, a possible involvement of the mammalian target of rapamycin (mTOR) signaling pathway, which has been shown to play a positive role in cell proliferation and survival [26], was investigated (Figure 6A). Down-regulation of the individual α- and α' subunits of CK2, respectively, resulted in decreased phosphorylation of p70 S6 kinase (p70S6K), a known downstream target of Raptor-mTOR [26], with respect to control experiment and cells treated with gemcitabine. The phosphorylation level of p70S6K did not further decrease when cells were additionally treated with gemcitabine. As mTOR is one of the effectors regulated *via *the phosphatidylinositol 3'-kinase (PI3K)/AKT signaling pathway, the expression and/or phosphorylation status of other PI3K/AKT pathway-related proteins were also investigated (Figure 6B). The expression of phosphoinositide-dependent protein kinase-1 (PDK1) and AKT protein kinases, both downstream effectors of PI3K, remained unchanged. In line with results shown in Figure 6A, the phosphorylation of AKT at S473, which is triggered by Rictor-mTOR complex [26], was also found decreased in cells depleted of CK2 subunits and in the absence and presence of gemcitabine, respectively (Figure 6B). Interestingly, while the down-regulation of CK2α resulted in a slight decrease of AKT phosphorylation at T308, lack of CK2α' expression led to a significant suppression of AKT phosphorylation at this residue which was accompanied by decreased phosphorylation of GSK3β. The addition of gemcitabine did not significantly modify the phosphorylation status of AKT observed in cells depleted of the sole CK2 catalytic subunits. The kinase activity of endogenous PDK1 was examined as T308 is one of the AKT-primary regulatory phosphorylation sites targeted by this kinase. Results indicated that the observed lack of phosphorylation of AKT at T308 in cells depleted of CK2α' was not a direct consequence of decreased activity of endogenous PDK1 (data not shown). Recently, it has been found that the CK2-mediated phosphorylation of AKT at S129, by increasing the association of heat shock protein 90 (Hsp90) to AKT, contributes to maintain a high T308 phosphorylation level [17,27]. Western blot analysis was performed in order to determine whether lack of phosphorylation of AKT at T308 in CK2α'-knockdown cells was caused by a reduced phosphorylation of AKT at S129. Results reported in Figure 6C, indicated that this was indeed the case. Overall, these results clearly show that not only the two catalytic subunits of CK2 contribute differently to enhance gemcitabine-induced cell death, with the reduced level of CK2α' being the most effective, but also that they exert a different effect on pro-survival signaling pathways in gemcitabine-resistant pancreatic cancer cells.

## Discussion

Gemcitabine-based therapy remains the first-line treatment for both locally advanced and metastatic pancreatic cancer and serves as the standard to which new treatment regimens are compared. In this study, the initial investigation of four pancreatic adenocarcinoma cell lines on the cellular response to increasing concentrations of gemcitabine revealed a variable degree of sensitivity towards drug treatment. Overall, while Capan-1 and BxPC-3 cell lines were the most affected by the treatment, PANC-1 cells showed high resistance towards gemcitabine with respect to viability and proliferation. Cellular depletion of the individual CK2 catalytic subunits in combination with gemcitabine resulted in enhanced cell death with respect to the sole gemcitabine treatment. Interestingly, down-regulation of CK2α' but not -α, was sufficient to kill the cells and the percentage of cell death slightly increased when gemcitabine was added suggesting that CK2α' may exert a unique function associated with the control of cell survival. Several studies reported the tendency of CK2α_2_β_2 _but not CK2α'_2_β_2 _to form aggregates suggesting that aggregation could be a mean to regulate CK2 activity, whereby the protomer would be the active form and the oligomer would be inactive [28,29]. As shown in Figure 3A (insert), the expression of CK2α' is significantly lower than the one of CK2α. Interestingly, following siRNA treatment against the individual subunits, a similar decrease in the kinase activity was observed. Hence, it is assumed that in PANC-1 cells CK2α'_2_β_2 _would be the prevalent soluble and active tetrameric form. This would explain the large effect on cell death seen in cells depleted of CK2α'. Nevertheless, it cannot be excluded that CK2α' might be present predominantly as a monomer. In this respect, evidence indicates that the monomeric form of CK2 seems to be more effective in phosphorylating AKT [17] and more implicated in cell survival and resistance to chemotherapeutic drug treatment [15]. The potential involvement of various protein kinases was determined and data indicate that gemcitabine treatment in cells lacking CK2α specifically leads to JNK phosphorylation suggesting that the JNK pathway, whose role in cell death is well established [30,31], might contribute to cell killing in pancreatic cancer cells through a cross-talk with CK2α.

Numerous studies have reported that the PI3K/AKT/mTOR signaling pathway is constitutively active in pancreatic cell lines [32-37]. Moreover, previous data showed that the gemcitabine-resistance mechanism in PANC-1 cells is associated with amplification of the gene coding for AKT [30,35]. Lack of expression of the CK2 catalytic subunits led to suppression of p70S6K and AKT phosphorylation at the regulatory T389 and S473 amino acid residues, respectively, suggesting that mTOR activity is impaired when CK2 expression is suppressed. While these results demonstrated a cross-talk between CK2 and mTOR, nevertheless they could not explain the different percentages of cell death achieved with down-regulation of CK2α and -α', respectively. Interestingly, down-regulation of CK2α' resulted in a significant decrease in the phosphorylation and activity of AKT at T308 which was confirmed by the lowered phosphorylation of GSK3β. The reported difference in the phosphorylation levels of AKT suggests that the modulation of AKT activity contributes to the different amounts of cell death observed following the aforementioned treatments. Given the fact that the activity of PDK1 did not vary, the mechanism by which lack of CK2α' resulted in suppression of AKT phosphorylation at T308 remained to be determined. Results reported here are consistent with previously published data on a direct involvement of CK2 in the phosphorylation of AKT at S129 which facilitates AKT binding to Hsp90 chaperone, thus preventing T308 dephosphorylation [27]. Indeed upon down-regulation of CK2α', we found that, the phosphorylation of AKT at S129 was significantly reduced.

## Conclusions

The findings presented here indicate a general cooperation between CK2 and the PI3K/AKT and MKK4/JNK pathways in promoting survival of pancreatic cancer cells. Modulation of expression of the individual CK2 catalytic subunits has various effects on the aforementioned signaling pathways. Moreover, the data suggest that inhibition [38] or suppression of CK2 ([14], present paper) are promising objectives of novel molecular targeting therapies for pancreatic cancer but given the complex biology of this type of malignancy, the simultaneous targeting of several survival pathways would certainly improve the chances of efficient tumor treatment and outcomes of patients with pancreatic cancer.

## Competing interests

The authors declare that they have no competing interests.

## Authors' contributions

JNK carried out most of the experiments reported in the manuscript, participated in the design of the paper and critically revised it, MR provided reagents and critically revised the manuscript, BG conceived the study, carried out some experiments and wrote the paper. All authors read and approved the final manuscript.

## Pre-publication history

The pre-publication history for this paper can be accessed here:

http://www.biomedcentral.com/1471-2407/10/440/prepub
